# Benefits of supplementation with multiple micronutrients in pregnancy

**DOI:** 10.1111/nyas.14088

**Published:** 2019-04-29

**Authors:** Robert E. Black, Kathryn G. Dewey

**Affiliations:** ^1^ Institute for International Programs and Department of International Health, Bloomberg School of Public Health Johns Hopkins University Baltimore Maryland; ^2^ Program in International and Community Nutrition and Department of Nutrition University of California Davis California

**Keywords:** nutrients, micronutrients, pregnancy, LMIC, folic acid, iron

## Abstract

A task force was convened by the New York Academy of Sciences to evaluate new evidence that was not available at the time of, and to help countries interpret, recent World Health Organization guidelines for nutrition interventions in pregnancy as they relate to multiple micronutrient supplementation. The report of the task force is published in a recent special issue of *Annals of the New York Academy of Sciences*. Here, we provide a short introduction to the special issue.

Good nutrition during pregnancy is important for fetal development and survival and for growth from birth through childhood. Inadequate maternal nutrition can result in infants having low birth weight (LBW, defined as birth weight of <2500 g), which is caused by preterm birth, fetal malnutrition, or both together. It is estimated that in 2015, 14.6% of births globally were LBW totaling 20.5 million births, mainly in South Asia and sub‐Saharan Africa.^1^ Babies with LBW, both those who are preterm or have fetal malnutrition, assessed as being born small for gestational age (SGA), as well as babies who are SGA and not LBW, have an elevated risk of death in infancy.^2^ In addition, these babies have an elevated risk of stunting of linear growth and development in childhood and of adult‐onset chronic diseases.^3^

Pregnant women in low‐ and middle‐income countries (LMICs) commonly have nutrient‐poor diets that result in deficiencies of multiple vitamins and minerals, collectively referred to as micronutrients. A large number of trials in pregnancy in these settings provided multiple micronutrient supplements (MMS) to determine if, in comparison with the usual supplement of iron and folic acid (IFA), there was a reduction in LBW or other adverse outcomes at birth or in the neonatal period. A systematic review of 15 trials, considered in the development of guidelines for nutrition interventions in pregnancy by the World Health Organization (WHO), found that newborns of mothers who received MMS were significantly less likely to be LBW or SGA.^4^ While the WHO 2016 antenatal care guidelines continued to recommend IFA supplements for routine use, they stated that “countries with a high prevalence of nutritional deficiencies might consider the benefits of MMS on maternal health to outweigh the disadvantages and may choose to give MMS that include iron and folic acid.”^5^ A task force was convened by the New York Academy of Sciences to evaluate new evidence that was not available at the time of the development of the guidelines and to help countries interpret the guidelines in relation to MMS in pregnancy. The report of the task force is published in a recent special issue of *Annals of the New York Academy of Sciences*.^6^

The task force found from a review of evidence that in women of reproductive age and pregnant women in LMIC, there is a high prevalence of deficiencies of multiple essential micronutrients. It also considered an individual participant data meta‐analysis of multiple micronutrient supplement trials that was published after the WHO antenatal guidelines.^7^ This analysis confirmed the findings of the previous meta‐analysis, including the observation that women receiving MMS, compared with those receiving only IFA, had a lower risk of LBW and SGA births. Additionally, it identified a number of subgroups that had greater benefits from MMS. Anemic women benefited more from MMS than nonanemic women in regard to LBW, SGA, and reduced fetal and infant deaths, and underweight women who received MMS had a reduction in preterm births. MMS did not increase the risk of stillbirth, neonatal, 6‐month, or infant mortality overall, or in the analysis of any subgroup, such as those based on maternal stature or nutritional status, compared with IFA. The WHO antenatal care guideline indicated a concern regarding a possible risk of increased neonatal mortality from MMS with 30 mg of iron compared with IFA with 60 mg of iron. However, a new analysis making this comparison with corrected and new data from several trials did not find a difference in the relative risk of neonatal deaths with this comparison.^8^

Based on the efficacy and safety data, the task force concluded that MMS provide greater benefit than IFA for birth outcomes and infant mortality and that the UNIMMAP formulation used in most of the controlled trials could be a basis for going forward in programs. Populations with a high prevalence of anemia and underweight in women of reproductive age could be prioritized for the use of this multiple micronutrient supplement. Additional data on the nutritional conditions, including micronutrient deficiencies, among women in LMIC would be useful to design and monitor supplementation programs. WHO antenatal care guidelines for use of IFA do not specify the number of days that supplementation should be given during pregnancy, but indicate that it should be started as early in pregnancy as possible. The evidence for MMS, showing that women who started before 20 weeks of gestation had a reduction in preterm births compared with no effect in those who started later,^7^ suggests that the same advice applies to MMS.

A second paper published in the special issue addresses the question of whether multiple micronutrient consumption might lead to excess intakes of certain micronutrients.^9^ For many micronutrients, there may be health risks associated with regular intake that exceeds an upper threshold for safety, typically called an upper level (UL). To examine this question, the intake of each nutrient that would result from consuming the UNIMMAP formulation daily, on top of a diet that already included the recommended intake of that nutrient, was compared with the UL. For most micronutrients, this combination was substantially below the UL. Only three nutrients met or exceeded the UL: folate, iron, and niacin. For folate, the total from both sources (diet and UNIMMAP) just met the UL. The main concern with regard to excess folate intake is the potential to mask vitamin B12 deficiency. Because MMS include vitamin B12, this risk is mitigated, which is not the case with IFA supplementation. In the case of iron, IFA and MMS both contain iron, so there is no additional risk of consuming MMS instead of IFA with regard to exceeding the UL for iron. The optimal dose of iron during pregnancy and the potential adverse effects of excess iron are still unclear, and this topic is a high priority for additional research.^10^ Meanwhile, the usual dose of iron included in MMS (30 mg/day) is less likely to lead to excess intake than doses of 60 mg or higher. For niacin, the UL is based on the occurrence of flushing, which is a very mild and temporary side effect. Moreover, the UL is based only on intake from synthetic forms of niacin from supplements and fortified foods, not from naturally occurring niacin in the diet, and the UNIMMAP formulation contains an amount (18 mg) that is well below the UL of 35 mg. Thus, the risk of multiple micronutrient consumption leading to excess intake of micronutrients appears to be low.

A third paper from the task force addresses a question raised in the WHO antenatal care guidelines regarding the cost‐effectiveness of MMS.^11^ The multiple micronutrient supplement has a small incremental cost compared with IFA because of the additional micronutrients. The switch from IFA to MMS was shown to be very cost‐effective in analyses for Bangladesh and Burkina Faso. The estimated cost of $3–15 per Disability Adjusted Life Year (DALY) averted for these countries compares favorably with other highly cost‐effective maternal and child interventions. For comparison, other interventions include micronutrient fortification (USD20–100 per DALY averted), balanced protein‐energy supplementation for women in impoverished households (USD500 per DALY averted), and Cesarean section (USD1600–2600 per DALY averted).^12^ Furthermore, the switch from IFA to MMS was estimated to have a cost of USD125 and USD184 per death averted for Burkina Faso and Bangladesh, respectively. This is highly cost‐effective and compares very favorably with packages for perinatal and newborn care, including midwifery and obstetric services, costing USD1000–3000 per death averted.^13^ The change to MMS has a low cost per case of LBW averted (USD37–44 in these two countries),^11^ but because there are few other effective interventions for this outcome, comparative cost‐effectiveness data are not available.

MMS in pregnancy are commonly used in the United States and other high‐income countries. Recognizing the nutritional needs of their populations and the benefits of MMS in pregnancy, a number of LMICs are introducing these supplements with support from UNICEF and other organizations. This will provide the opportunity to learn how to assure a high‐quality supply of MMS, availability during antenatal care or other channels, and adherence throughout pregnancy, as well as to identify methods to target populations at high risk of poor birth outcomes because of nutritional deficiencies. It is important that MMS become part of more comprehensive and integrated health care and nutritional support for women before, during, and after pregnancy and for newborns, infants, and children so that any benefits achieved at birth can be sustained throughout the life course.

## Competing interests

The authors declare no competing interests.
